# Nurses and University Training

**Published:** 1919-07-05

**Authors:** 


### THE MATRONS' AND SISTERS' DEPARTMENT.

### NURSES AND UNIVERSITY TRAINING.

A great deal of interest and enthusiasm was roused among members of the College of Nursing by Miss Cox-Davies's remarks at the annual meeting relating to a Chair of Nursing. It was a great step on the road to higher education when the College instituted scholarships to be competed for by trained nurses. It would be another and greater advance to plant the banner of nursing in a women's college and secure the continual presentment of the highest nursing ideals in the environment chosen by women of intellect for the completion of their education.

Although the Chair of Nursing is at the moment but a project, it contains within itself the germ of so much which is of value to this generation that it cannot fail to be realised before very long. There is but one objection to the founding of a Professorship of Nursing. It recognises that there is a large field of study desirable for the nurse outside the sick-room or ward. Hitherto nurses have acquired their art side by side with their duties towards the sick, passing from class and lecture to take their share in the labours of the hospital always strongly penetrated with the sense that if study and ward work clash, it is the former which must give way. There are many earnest-minded nurses who depreciate the side of nursing which is divorced from contact with the sick. Yet more and more clearly the modern hospital system demands from those who would take a share in directing the nursing a wide study of matters not to be found within the compass of ward work. Either these things must be taught to pupils burdened with the weight of ward duties, or they must be taught before or after the training as a nurse has taken place. The modern tendency is for some things to be taught before training and some after, and the establishment of a Chair of Nursing would help to systematise this tendency.

It is probable that attendance at the course of nursing which it is hoped to arrange in one of the women's colleges would need to meet the requirements of two classes of students, those of nurses who had completed three or four years' training in hospital, and those of women who, while enjoying a period of academic life after leaving school, were drawn to study nursing by the opportunity presented to them in college, as a preliminary to taking formal training in hospital. Undoubtedly it would be preferable for women to take the hospital training before the collegiate. It would simplify the labours of the lecturer, and obviate the slightest danger of any woman thinking that lectures alone could make her a nurse. At the same time, to shut the door in the face of lay women who were drawn to the study of nursing, by excluding all who were not already trained, would mean the loss to the profession of many who would do it great credit, for the study of nursing is the mother of vocation. The nursing profession needs more intellectual women, and has within it a compelling power which would surely bring into the true fold eventually those who had begun their apprenticeship with theory instead of with practice. Probably this class of student could best be dealt with by dividing the course and admitting to the final part, which would qualify for a nursing diploma, only such candidates as had taken the prescribed course of training in hospital.

It will be in the highest degree interesting to watch the development of the higher nursing qualifications. The old-fashioned notion that a rather thicker layer than usual of elementary anatomy and physiology sums up the nurse's higher education has long been swept away. Every one now recognises that there are many subjects needing advanced study if the nurse is to realise the ideal of Florence Nightingale and become mistress of the entire environment of the sick and of the healthy and qualify herself to be the trained handmaid of those who lead the way in prevention as well as in cure. In nursing ethics alone, as Miss Lloyd Still has lately reminded us, there is a large field awaiting cultivation. In public health are vast problems which await the study of nurses dedicated to their myriad phases. Domestic science subjects, the knowledge of food properties, that of catering for large numbers, and of controlling establishments demand long preparation and study; they can only be superficially mastered by women who learn their business during three busy months spent in the housekeeper's department of a big hospital. There is the art of teaching and speaking, most neglected of all in our hospital training, without which no nurse can make her mark. And there is the history of nursing. It is clear that the first occupant of the Chair of Nursing will not find it easy to make a choice from among the numerous subjects which press for inclusion in the curriculum.

